# Lead Exposure and Tremor among Older Men: The VA Normative Aging Study

**DOI:** 10.1289/ehp.1408535

**Published:** 2015-01-23

**Authors:** John S. Ji, Melinda C. Power, David Sparrow, Avron Spiro, Howard Hu, Elan D. Louis, Marc G. Weisskopf

**Affiliations:** 1 Department of Environmental Health, and; 2 Department of Epidemiology, Harvard T.H. Chan School of Public Health, Boston, Massachusetts, USA; 3 VA Boston Healthcare System, Boston, Massachusetts, USA; 4 School of Public Health, and; 5 School of Medicine, Boston University, Boston, Massachusetts, USA; 6 Dalla Lana School of Public Health, University of Toronto, Toronto, Ontario, Canada; 7 Mailman School of Public Health, Columbia University, New York, New York, USA

## Abstract

Background: Tremor is one of the most common neurological signs, yet its etiology is poorly understood. Case–control studies suggest an association between blood lead and essential tremor, and that this association is modified by polymorphisms in the δ-aminolevulinic acid dehydrogenase (*ALAD*) gene.

Objective: We aimed to examine the relationship between lead and tremor, including modification by *ALAD*, in a prospective cohort study, using both blood lead and bone lead—a biomarker of cumulative lead exposure.

Methods: We measured tibia (*n* = 670) and patella (*n* = 672) bone lead and blood lead (*n* = 807) among older men (age range, 50–98 years) in the VA Normative Aging Study cohort. A tremor score was created based on an approach using hand-drawing samples. *ALAD* genotype was dichotomized as *ALAD-2* carriers or not. We used linear regression adjusted for age, education, smoking, and alcohol intake to estimate the associations between lead biomarkers and tremor score.

Results: In unadjusted analyses, there was a marginal association between quintiles of all lead biomarkers and tremor scores (*p*-values < 0.13), which did not persist in adjusted models. Age was the strongest predictor of tremor. Among those younger than the median age (68.9 years), tremor increased significantly with blood lead (*p* = 0.03), but this pattern was not apparent for bone lead. We did not see modification by *ALAD* or an association between bone lead and change in tremor score over time.

Conclusion: Our results do not strongly support an association between lead exposure and tremor, and suggest no association with cumulative lead biomarkers, although there is some suggestion that blood lead may be associated with tremor among the younger men in our cohort.

Citation: Ji JS, Power MC, Sparrow D, Spiro A III, Hu H, Louis ED, Weisskopf MG. 2015. Lead exposure and tremor among older men: the VA Normative Aging Study. Environ Health Perspect 123:445–450; http://dx.doi.org/10.1289/ehp.1408535

## Introduction

Tremor is one of the most commonly encountered neurological signs, and it may be a feature of a variety of neurological diseases. Action tremor, which occurs with voluntary movement, is the hallmark feature of essential tremor (ET), which is considered one of the most prevalent neurological diseases, and prevalence increases with age. Despite the ubiquity of tremor among the elderly, there are limited data on the role of environmental exposures that are potentially modifiable. Lead is one environmental neurotoxicant that has been suspected as a risk factor for ET (Louis 2008).

Case–control studies in two settings have examined the association between elevated blood lead concentration and ET (Centers for Disease Control and Prevention 2014; Dogu et al. 2007; Louis et al. 2003, 2005): one based in the New York metropolitan area and the other in Mersin, Turkey. In each setting, the risk of ET was found to be higher with higher blood lead concentrations. In New York, a strong modification by a polymorphism in the δ-aminolevulinic acid dehydrogenase (*ALAD*) gene was seen (Louis et al. 2005). However, blood lead has a half-life of approximately 30 days (Hu et al. 1998; Rabinowitz 1991) and is more a biomarker of recent exposure than of chronic exposure to environmental lead. If lead-induced neurotoxicity results in ET, or more broadly in action tremor, it is likely chronic exposure may be more relevant.

To examine the association between chronic lead exposure and action tremor, we used data from participants in the Department of Veterans Affairs (VA) Normative Aging Study (NAS), a cohort of community-dwelling elderly men in the Greater Boston, Massachusetts, area with measures of lead in bone (a biomarker of cumulative lead exposure) and blood (a biomarker of recent exposure) and measures of tremor.

## Methods

*Study sample*. Our study sample is a subgroup of the NAS, a longitudinal study of aging established by the VA in 1963 when 2,280 men from the Greater Boston area who were 21–80 years of age were enrolled (Bell et al. 1966). Men with a history of treatment for hypertension, systolic blood pressure > 140 mmHg, diastolic blood pressure > 90 mmHg, or other chronic conditions, including heart disease, diabetes mellitus, and cancer, were not admitted into the study. NAS subjects have reported for medical examinations every 3–5 years, at which data on smoking history, education level, food intake, and other risk factors that may influence health were also collected. Cognitive testing results have been collected since 1993. The human research committees of the VA, Partners Health Care, and Harvard School of Public Health approved this research, and written consent was obtained from all participants.

Of 1,287 active NAS participants when bone lead measurements began in 1991, 878 (68%) gave their consent for K-shell X-ray fluorescence (KXRF) measurements of lead concentration in both tibia and patella bone. NAS participants with KXRF measurements were similar to those without them with respect to distributions of age, race, education, smoking status, consumption of alcohol, and retirement status, although they tended to have slightly higher blood lead concentrations (Hu et al. 1996). We excluded individuals with patella measurement uncertainty > 15 μg/g (*n* = 3) and tibia measurement uncertainty > 10 μg/g (*n* = 11) because these usually reflect excessive subject movement during the measurement. We also excluded nonwhites (*n* = 19) because there were too few to consider separately. Diagnoses of ET were not assigned in NAS; however, it was possible to assess action tremor because participants produced drawings as part of cognitive tests that required them to copy different figures. These figure-drawing samples were completed a median of 13 (interquartile range, 41–0) days before the bone lead measurements. Of those with valid bone lead measures, 672 subjects with a patella lead measurement and 670 subjects with a tibia lead measurement also completed the cognitive testing that included figure copying. Blood was collected from virtually all participants at their regularly scheduled VA clinic visits and was used to measure blood lead concentration. We analyzed data from the 807 NAS participants who had blood drawn (used for lead measurement) on the same day as they completed the figure copying.

*Blood lead assessment*. Blood lead samples were collected in a special lead-free tube containing EDTA. Blood samples were sent to ESA Laboratories (Chelmsford, MA) for analysis by Zeeman background-corrected flameless atomic absorption (graphite furnace) calibrated with National Institute of Standards and Technology (NIST) Blood Lead Standard Reference Materials. Ten percent of the samples were run in duplicate, 10% were standards, and 10% were blanks, analysis of which produced no evidence of external contamination or significant problems with reliability. In tests on reference samples from the Centers for Disease Control and Prevention, the coefficients of variation were 1–8%.

*Bone lead assessment*. Bone lead concentration measurements were taken with a KXRF instrument (ABIOMED, Danvers, MA) at both the mid-tibia shaft (midpoint between the tibial plateau and the medial malleolus) and patella while the subject was seated. Mid-tibia (shin bone) is composed primarily of cortical bone with a lead half-life of many decades; patella (knee cap bone) is primarily trabecular bone and has a lead half-life of a few years (Wilker et al. 2011). Thirty-minute measurements were taken at each site, after the skin at each measurement site had been washed with a 50% solution of isopropyl alcohol. The KXRF beam collimator was sited perpendicular to the flat bony surface of the tibia and at 30° in the lateral direction for the patella.

ALAD *genotype assessment*. Previous studies suggest effect modification by *ALAD* genotype (Louis et al. 2005). The *ALAD* gene is located on chromosome 9q32, and the reference SNP (single nucleotide polymorphism) identification number for the polymorphism is rs1800435. We determined participants’ *ALAD* genotype through amplifications on 0.5 μL of whole blood using two sets of primers specific for a portion of the *ALAD* gene. Primer sequences for the initial round of amplification were (5´-AGAC​AGAC​ATTA​GCTC​AGTA-3´) and (5´-GGCA​AAGA​ACAC​GTCC​ATTC-3´). Amplified DNA was then restricted using *MSP*I and electrophoresed through 2.0% agarose. *ALAD* alleles are differentiated based on the existence of a *MSP*I endonuclease restriction site specific to the *ALAD-2*–derived PCR (polymerase chain reaction) fragment, which yields a diagnostic restriction band. Heterozygotes exhibit both the *ALAD-1* and *ALAD-2* fragments and can thus be differentiated from homozygotes of either type. We performed PCR reactions in duplicate, with blank controls included in each set (Bradley and Mash 2009). We combined *ALAD* 1-2 and 2-2 groups as a single indicator variable and used *ALAD* 1-1 as the reference group (Caller et al. 2012).

*Tremor assessment*. Numerical ratings of tremor were assigned based on hand-drawn samples as described in previous studies (Hoffman et al. 2012; Vieira et al. 2012). These scores represent action tremor, which we hereafter refer to simply as tremor. The hand-drawn samples were derived from figure-copying testing performed as part of a larger cognitive test battery. The copying included items from the Consortium to Establish a Registry for Alzheimer’s Disease (CERAD) battery, Mini-Mental State Examination (MMSE), and developmental test of visual–motor integration (VMI) (Welsh et al. 1994). A research assistant was trained in-person by a senior movement disorders neurologist who specializes in tremor disorders (E.D.L.) to rate the severity of tremor based on eight drawings of figures. Visual examples from 25 NAS participants with a range of tremor severity were used for this 4-hr in-person training; thus 200 drawings were co-rated and discussed in detail. Tremor on each figure was rated using an ordinal scale: 0 (no tremor), 0.5 (possible tremor), 1 (clear tremor that was mild), 1.5 (mild to moderate tremor), 2 (moderate tremor). Based on the eight rated items, a total tremor score was generated for each subject by summing the scores for each of the eight figures (possible range, 0–16). During the course of subsequent post-training ratings, interrater agreement was high (Pearson correlation, 0.82), based on 20% of samples randomly selected for independent co-rating by the rater and the neurologist to assess interrater agreement in total tremor scores. Based on previously established cut-off points (Hoffman et al. 2012), drawing samples from participants whose total tremor score was ≥ 4.5 were sent to the senior neurologist (E.D.L.), who made a final determination of whether or not the tremor was sufficient to be considered moderate tremor in the range of essential tremor, which we refer to hereafter as elevated tremor; this assignment was based on the presence of clear tremor of moderate amplitude in two or more drawings in the absence of medications that could induce tremor (e.g., lithium, valproate), Parkinson’s disease by history, or hyperthyroidism by history.

*Statistical methods*. We used linear regression to assess the association between lead biomarkers, categorized into quintiles, and continuous tremor scores in our primary analyses. The significance of the trend was assessed by assigning to each person the median of their lead biomarker quintile, and using this variable to test for linear trends. This approach reduces the influences of extreme exposure values. Covariates considered in the models taken from the regular NAS questionnaires included age (years), age squared, alcohol (none and tertiles of consumption), pack-years of smoking (nonsmokers and tertiles among smokers), and education level (less than high school, high school graduate, less than college, and college graduate and beyond college). In secondary analyses we considered the dichotomous outcome of elevated tremor defined by the study neurologist, and used logistic regression to estimate the odds ratio (OR) and 95% confidence interval (CI) by levels of the lead biomarkers. We also conducted several sensitivity analyses. First, the analyses described above were repeated with lead biomarkers treated as continuous variables, both as untransformed and after log transformation. Second, we evaluated whether additional adjustment for *ALAD* status or medications, specifically self-reported use of beta-blockers (AHFS Pharmacologic-Therapeutic Classification code 24:24; http://www.ahfsdruginformation.com/pt-classification-system.aspx) and calcium channel blockers (AHFS Pharmacologic-Therapeutic Classification code 24:28) at the time of tremor assessment, affected our results. Beta-blockers such as propranolol and primidone are first-line treatments for ET, and can produce a reduction in tremor (Murch et al. 2004; Pablo et al. 2009). Calcium channel blockers, such as nifedipine, are used to treat high blood pressure and may also affect tremor (Alonso et al. 2010). Because < 5% of the study population reported using anticonvulsants, thyroid agents, or antiparkinsonian agents, we chose not to consider these medications. Additionally, using stratified analyses, we evaluated whether there was an effect of lead, categorized into quintiles, on tremor, treated as a continuous variable, in subgroups defined by *ALAD* and use of beta-blockers and/or calcium channel blockers. Because many more factors may contribute to tremor in older age, making the detection of a subtle environmental contributor more difficult to detect, we also examined the association between lead and tremor score separately among those below or at and above the median age (68.9 years).

Finally, using the most recently available figure-copying samples from subsequent follow-up assessments of the NAS participants, we were able to obtain a second assessment of tremor (mean ± SD = 8.0 ± 3.2 years after the first assessment) in 427 participants with bone lead assessments who had an initial tremor score of < 4.5. We then used linear regression to estimate the association between quintile of lead biomarker data and change in tremor score in this group. All analyses were performed using SAS version 9.2 (SAS Institute Inc., Cary, NC).

## Results

The men in our study ranged in age from 50 to 98 years, with mean (± SD) of 69.0 ± 7.2 years. Overall, the mean blood lead concentration was 5.0 ± 2.7 μg/dL, and the mean tibia and patella bone lead were 21.2 ± 13.3 and 28.0 ± 18.4 μg/g, respectively. The medians for blood, tibia, and patella were 4.0 μg/dL, 19.0 μg/g, and 24.0 μg/g, respectively. The correlations between lead measurements were high (Spearman correlation = 0.66 for patella and tibia; 0.40 for both patella and blood; 0.38 for tibia and blood). The distribution of lead concentrations by different characteristics of the study sample is shown in Table 1. As has been reported previously (Hu et al. 1996), lead concentrations increase with higher age, lower education, and more smoking.

**Table 1:** Lead biomarker and tremor scores by demographic characteristics of study population.

Characteristic	Blood lead			Patella lead			Tibia lead		
	*n*	Mean ± SD(μg/dL)	Tremor score(mean ± SD)	*n*	Mean ± SD (μg/g)	Tremor score (mean ± SD)	*n*	Mean ± SD(μg/g)	Tremor score (mean ± SD)
Total	807	5.01 ± 2.72	3.96 ± 1.01	672	27.98 ± 18.38	3.99 ± 0.98	670	21.23 ± 13.29	3.99 ± 0.99
Age (years)
< 55	16	3.88 ± 2.06	3.72 ± 0.48	12	12.67 ± 4.89	3.63 ± 0.48	12	10.50 ± 5.57	3.63 ± 0.48
55–59	63	4.30 ± 1.92	3.67 ± 0.83	48	18.79 ± 11.31	3.71 ± 0.71	48	12.28 ± 6.74	3.64 ± 0.85
60–64	176	4.82 ± 2.51	3.89 ± 0.97	143	23.33 ± 11.29	3.92 ± 0.96	143	18.28 ± 10.16	3.92 ± 0.96
65–69	190	5.07 ± 2.49	3.92 ± 0.89	172	27.71 ± 18.51	3.94 ± 0.83	174	20.41 ± 12.32	3.94 ± 0.83
70–74	206	5.14 ± 2.85	3.87 ± 0.82	170	31.62 ± 22.15	3.88 ± 0.78	168	24.28 ± 15.11	3.88 ± 0.78
75–79	98	5.28 ± 3.63	4.29 ± 1.37	85	31.07 ± 17.61	4.34 ± 1.28	82	23.52 ± 11.19	4.34 ± 1.30
80–84	45	5.58 ± 2.70	4.16 ± 0.89	34	39.76 ± 21.85	4.26 ± 0.72	35	31.91 ± 19.05	4.20 ± 0.81
≥ 85	13	5.46 ± 2.26	5.65 ± 2.14	8	34.61 ± 11.47	6.31 ± 2.49	8	27.30 ± 13.33	6.31 ± 2.49
Education
Missing	33	4.67 ± 2.15	3.88 ± 0.99	26	27.84 ± 19.30	3.83 ± 0.62	26	18.13 ± 9.82	3.83 ± 0.62
College graduate and beyond	211	4.64 ± 2.31	4.00 ± 1.18	192	22.93 ± 13.22	4.04 ± 1.18	191	17.10 ± 10.12	4.04 ± 1.19
Less than college	203	4.65 ± 2.40	3.90 ± 1.10	167	25.79 ± 16.08	3.97 ± 1.05	167	20.90 ± 11.30	3.94 ± 1.09
High school graduate	283	5.38 ± 3.21	3.97 ± 0.81	222	31.59 ± 20.88	3.99 ± 0.76	223	23.52 ± 14.93	3.98 ± 0.76
Less than high school	77	5.74 ± 2.60	4.04 ± 1.00	65	36.21 ± 22.25	4.01 ± 0.90	63	27.77 ± 17.15	4.03 ± 0.91
Alcohol consumption
Nondrinker	211	4.74 ± 2.28	4.07 ± 1.16	180	28.46 ± 17.06	4.06 ± 1.17	178	22.47 ± 12.44	4.06 ± 1.17
Tertile 1 of drinkers	197	4.41 ± 2.42	3.89 ± 0.96	160	24.68 ± 16.91	3.94 ± 0.93	161	19.75 ± 12.54	3.93 ± 0.94
Tertile 2 of drinkers	194	5.14 ± 3.05	3.87 ± 0.89	163	28.57 ± 21.28	3.90 ± 0.78	161	21.47 ± 15.35	3.88 ± 0.83
Tertile 3 of drinkers	191	5.75 ± 2.98	4.03 ± 0.98	162	30.18 ± 17.96	4.06 ± 0.94	163	21.14 ± 12.46	4.06 ± 0.93
Missing	14	5.36 ± 2.27	3.71 ± 1.40	7	26.00 ± 14.06	4.14 ± 1.41	7	20.14 ± 18.72	4.14 ± 1.41
Smoking
Nonsmoker	218	4.90 ± 2.62	3.98 ± 1.11	196	25.57 ± 16.21	4.04 ± 0.92	201	19.74 ± 12.43	4.00 ± 0.96
Tertile 1 of pack-years	187	4.75 ± 2.89	3.89 ± 0.84	160	25.61 ± 16.58	3.86 ± 0.87	158	20.75 ± 12.63	3.86 ± 0.87
Tertile 2 of pack-years	201	4.88 ± 2.24	3.86 ± 0.93	152	27.00 ± 16.36	3.92 ± 0.92	151	20.44 ± 11.07	3.93 ± 0.92
Tertile 3 of pack-years	189	5.52 ± 3.06	4.12 ± 1.14	153	34.27 ± 22.77	4.15 ± 1.19	150	24.32 ± 16.21	4.15 ± 1.20
Missing	12	5.08 ± 3.12	4.08 ± 0.51	11	31.18 ± 20.42	4.05 ± 0.57	10	24.30 ± 16.95	4.00 ± 0.58
ALAD
1-1	684	5.09 ± 2.80	3.99 ± 1.04	564	28.07 ± 18.73	4.00 ± 1.00	561	21.24 ± 13.75	3.99 ± 1.02
1-2 and 2-2	123	4.55 ± 2.18	3.84 ± 0.86	108	27.47 ± 16.50	3.99 ± 0.83	109	21.17 ± 10.69	3.99 ± 0.83

Tremor ratings among the 807 participants with blood lead measurements ranged from 0.5 to 11.5, with a mean (± SD) of 3.96 ± 1.01, median of 4.0, and an interquartile range of 3.5–4.0. As has been reported previously, we found that tremor scores increased with age, and this persisted when adjusted for education, smoking, and alcohol consumption (Louis 2008). In adjusted linear regression analyses, tremor score increased 0.25 points per 10 years of age (*p*-value < 0.001). In unadjusted analyses, there was a marginal association between quintiles of all lead biomarkers and total tremor scores (*p*-values = 0.11 for blood, 0.07 for patella, 0.13 for tibia), yet in adjusted models, we did not find an association between quintiles of lead biomarkers and total tremor scores (Table 2). In unadjusted analyses, there was only a marginal association between having elevated tremor and quintile of tibia lead, but this association was not significant in adjusted analyses (Table 3). The results were similarly null when lead measures were analyzed as continuous variables either untransformed or log transformed, after additional adjustment for *ALAD* genotype or use of beta-blockers and/or calcium channel blockers, and in subgroups defined by *ALAD* genotype or beta-blocker/calcium channel blocker medication use (data not shown).

**Table 2:** Difference in tremor scores by lead biomarker quintiles.

Linear regression	Quintile 1	Quintile 2	Quintile 3	Quintile 4	Quintile 5	*p*-Trend
Blood lead
*n*	244	178	122	153	110
Tremor score (mean ± SD)	3.89 ± 1.02	3.96 ± 1.10	3.98 ± 0.74	4.01 ± 1.03	4.06 ± 1.11
Lead range (μg/dL)	0 to 3	4 to 4	5 to 5	6 to 7	8 to 28
Crude model (95% CI)	0 (reference)	0.07 (–0.13, 0.26)	0.09 (–0.13, 0.31)	0.12 (–0.08, 0.33)	0.18 (–0.05, 0.41)	0.11
Adjusted^*a*^ model (95% CI)	0 (reference)	0.09 (–0.10, 0.29)	0.06 (–0.16, 0.28)	0.07 (–0.13, 0.27)	0.07 (–0.16, 0.30)	0.62
Patella lead
*n*	154	129	127	131	131
Tremor score (mean ± SD)	3.97 ± 1.01	3.98 ± 1.05	3.93 ± 0.68	3.87 ± 0.88	4.22 ± 1.16
Lead range (μg/g)	–9 to 15	16 to 21	22 to 28	29 to 39	40 to 165
Crude model (95% CI)	0 (reference)	0.01 (–0.22, 0.23)	–0.04 (–0.27, 0.19)	–0.10 (–0.33, 0.12)	0.24 (0.02, 0.47)	0.07
Adjusted^*a*^ model (95% CI)	0 (reference)	–0.07 (–0.30, 0.15)	–0.14 (–0.37, 0.09)	–0.22 (–0.45, 0.01)	–0.02 (–0.27, 0.22)	0.67
Tibia lead
*n*	142	124	136	133	135
Tremor score (mean ± SD)	3.88 ± 1.02	3.92 ± 1.06	4.00 ± 0.95	4.11 ± 0.98	4.02 ± 0.93
Lead range (μg/g)	–1 to 11	12 to 16	17 to 21	22 to 29	30 to 126
Crude model (95% CI)	0 (reference)	0.04 (–0.20, 0.28)	0.12 (–0.11, 0.36)	0.23 (–0.01, 0.46)	0.14 (–0.10, 0.37)	0.13
Adjusted^*a*^ model (95% CI)	0 (reference)	0.03 (–0.20, 0.26)	0.03 (–0.20, 0.26)	0.13 (–0.11, 0.36)	–0.07 (–0.32, 0.17)	0.70
^***a***^Adjusted for age, age squared, alcohol intake, cigarette pack-years, and education level.						

**Table 3:** Odds ratio (OR) for elevated tremor by lead biomarker quintiles.

Logistic regression	Quintile 1	Quintile 2	Quintile 3	Quintile 4	Quintile 5	*p*-Trend
Blood lead
Case/noncases (*n*)	24/220	16/162	12/110	17/136	11/99
Lead range (μg/dL)	0 to 3	4 to 4	5 to 5	6 to 7	8 to 28
Crude OR (95% CI)	Reference	0.91 (0.47, 1.76)	1.00 (0.48, 2.08)	1.15 (0.59, 2.21)	1.02 (0.48, 2.16)	0.80
Adjusted^*a*^ OR (95% CI)	Reference	0.96 (0.49, 1.90)	0.87 (0.40, 1.85)	1.03 (0.52, 2.04)	0.84 (0.38, 1.86)	0.72
Patella lead
Case/noncases (*n*)	10/144	14/115	16/111	13/118	13/118
Lead range (μg/g)	–9 to 15	16 to 21	22 to 28	29 to 39	40 to 165
Crude OR (95% CI)	Reference	1.75 (0.75, 4.09)	2.08 (0.91, 4.75)	1.59 (0.67, 3.75)	1.59 (0.67, 3.75)	0.51
Adjusted^*a*^ OR (95% CI)	Reference	1.51 (0.62, 3.64)	1.57 (0.66, 3.75)	1.29 (0.52, 3.21)	0.83 (0.31, 2.19)	0.41
Tibia lead
Case/noncases (*n*)	13/129	8/116	12/124	12/121	19/116
Lead range (μg/g)	–1 to 11	12 to 16	17 to 21	22 to 29	30 to 126
Crude OR (95% CI)	Reference	0.68 (0.27, 1.71)	0.96 (0.42, 2.19)	0.98 (0.43, 2.24)	1.63 (0.77, 3.44)	0.09
Adjusted^*a*^ OR (95% CI)	Reference	0.63 (0.25, 1.62)	0.84 (0.36, 1.99)	0.79 (0.33, 1.89)	1.08 (0.46, 2.53)	0.60
^***a***^Adjusted for age, age squared, alcohol intake, cigarette pack-years, and education level.						

Among those younger than the median (68.9 years), tremor score increased significantly with increasing quintile of blood lead (*p* = 0.03) with those in the highest quintile scoring 0.35 (95% CI: 0.03, 0.67) points higher than those in the lowest quintile (Table 4). This pattern was not apparent for either bone lead biomarker. Among those at least as old as the median age, there was no association between any lead biomarker and total tremor score.

**Table 4:** Adjusted^*a*^ difference in total tremor score by lead biomarker quintiles separately by age (years).

Exposure	Lead range	Mean difference (95% CI)	
		< Median age (68.9)	≥ Median age (68.9)
Blood lead (μg/dL)
Quintile 1	0 to 3	Reference	Reference
Quintile 2	4 to 4	0.12 (–0.12, 0.36)	0.08 (–0.23, 0.39)
Quintile 3	5 to 5	0.13 (–0.16, 0.42)	–0.04 (–0.38, 0.29)
Quintile 4	6 to 7	0.17 (–0.10, 0.44)	–0.03 (–0.34, 0.27)
Quintile 5	8 to 28	0.35 (0.03, 0.67)	–0.19 (–0.54, 0.15)
*p-*Trend		0.03	0.20
Patella lead (μg/g)
Quintile 1	–9 to 15	Reference	Reference
Quintile 2	16 to 21	–0.15 (–0.42, 0.13)	0.04 (–0.34, 0.42)
Quintile 3	22 to 28	–0.09 (–0.38, 0.20)	–0.20 (–0.57, 0.17)
Quintile 4	29 to 39	–0.27 (–0.55, 0.02)	–0.17 (–0.56, 0.21)
Quintile 5	40 to 165	0.05 (–0.32, 0.42)	0.02 (–0.35, 0.38)
*p-*Trend		0.61	0.88
Tibia lead (μg/g)
Quintile 1	–1 to 11	Reference	Reference
Quintile 2	12 to 16	0.04 (–0.23, 0.31)	–0.06 (–0.47, 0.35)
Quintile 3	17 to 21	0.21 (–0.08, 0.50)	–0.23 (–0.61, 0.15)
Quintile 4	22 to 29	0.12 (–0.18, 0.43)	–0.02 (–0.39, 0.36)
Quintile 5	30 to 126	0.12 (–0.24, 0.47)	–0.24 (–0.61, 0.14)
*p-*Trend		0.37	0.31
^***a***^Adjusted for age, age squared, alcohol intake, cigarette pack-years, and education level.			

Finally, we also analyzed the change in tremor scores from baseline assessment to assessments an average of 8.0 ± 3.2 years after bone lead measurement. Among NAS men who scored < 4.5 on the first tremor assessment, we found no association between bone lead biomarkers and change in tremor score (Table 5).

**Table 5:** Change in total tremor scores in follow-up assessment where first tremor score is below 4.5, by lead biomarker quintiles.

Linear regression	Quintile 1	Quintile 2	Quintile 3	Quintile 4	Quintile 5	*p*-Trend
Patella lead
*n*	114	86	76	78	73
Range (μg/g)	–9 to 15	16 to 21	22 to 28	29 to 39	40 to 165
Crude model (95% CI)	0 (reference)	0.02 (–0.31, 0.36)	0.07 (–0.27, 0.42)	0.06 (–0.28, 0.41)	0.10 (–0.25, 0.45)	0.56
Adjusted^*a*^ model (95% CI)	0 (reference)	0.06 (–0.28, 0.41)	0.09 (–0.27, 0.45)	0.06 (–0.30, 0.43)	0.12 (–0.28, 0.53)	0.59
Tibia lead
*n*	105	86	80	76	80
Range (μg/g)	–1 to 11	12 to 16	17 to 21	22 to 29	30 to 126
Crude model (95% CI)	0 (reference)	–0.03 (–0.37, 0.31)	–0.25 (–0.59, 0.09)	–0.18 (–0.54, 0.17)	0.05 (–0.29, 0.40)	0.93
Adjusted^*a*^ model (95% CI)	0 (reference)	–0.02 (–0.36, 0.33)	–0.27 (–0.63, 0.09)	–0.18 (–0.55, 0.18)	0.06 (–0.33, 0.45)	0.96
^***a***^Adjusted for age, age squared, alcohol intake, cigarette pack-years, and education level.						

## Discussion

In this cohort of community-dwelling elderly men, although unadjusted analyses were marginally significant, we did not find a significant overall association between blood or bone lead exposure biomarkers and ratings of action tremor or occurrence of elevated tremor after adjustment for potential confounders. Nor did we find this association to be modified by polymorphisms in the *ALAD* gene or by medication use. However, in an analysis that stratified by age, a significant association between higher blood lead concentration and worse tremor was seen among the younger half of NAS men in our study sample after adjustment for confounders, although this was weaker for bone lead. We found no association between bone lead and change in tremor over an average of 8 years of follow-up.

Older men are likely to have many more factors that contribute to tremor, including age itself as well as some medications and accumulating comorbidities that can affect tremor, such as hyperthyroidism. These other factors could obscure an association with lead and explain why we found an association only among the younger men. Alternatively, because participation in a cohort and follow-up within a cohort is often greater among healthier individuals (Alonso et al. 2009; Mein et al. 2012)—and absence of chronic conditions or their symptoms was an entry criterion for the NAS—this could have resulted in some selection of NAS participants and the subsample in our analysis that are less sensitive to the effects of lead. People more sensitive to lead would have been more likely to exhibit conditions that would either exclude them from participation or at least make them less likely to participate or continue to participate. This selection would be expected to be stronger among older individuals because more of their contemporaries would be showing such symptoms and conditions. Thus, we may see stronger associations among the younger half of our sample because the younger half is less selected for insensitivity to lead effects.

Because we *a priori* expected that cumulative lead exposure would be a stronger predictor of tremor than recent exposure, the lack of association with bone lead—a much better biomarker of cumulative exposure than blood lead—is puzzling. A concern with such a pattern of results is that it suggests that the association could be driven by reverse causation, whereby some aspect of tremor results in more bone loss and, thus, increased blood lead as lead is released from bone stores. However, no data suggest that persons with tremor or ET lose bone more rapidly than those without. One possibility is that lead-related effects contribute to tremor only if some other necessary event has occurred, at which point the lead-related effects—for example, neuroinflammation as a result of lead-induced oxidative stress—come into play with more immediate consequences. It is also possible that current lead burden acting via acute effects on the nervous system promotes tremor, rather than longer-term neurotoxic effects of lead. We also cannot rule out chance as an explanation for this result.

There is a limited literature exploring risk factors for tremor generally or for ET more specifically, and lead exposure in particular. The prior studies differed from the present study in that they considered clinical ET in a case–control study design with only blood lead as a measure of exposure (Dogu et al. 2007; Louis et al. 2003, 2005). In a New York study, 100 ET patients were identified from a computer database at a medical center in New York, and 143 controls were identified using random-digit dialing in the New York City metropolitan area and matched on age strata, sex, and ethnicity (Louis et al. 2003). Blood lead concentration was higher in the ET patients (mean, 3.3 ± 2.4 μg/dL) than in controls (mean, 2.6 ± 1.6 μg/dL). For each unit change of blood lead, the adjusted OR for ET was 1.19 (95% CI: 1.05, 1.39). In a hospital-based study in Mersin, Turkey, 105 ET cases were selected randomly from registration codes, and 105 controls were recruited from spouses or relatives of the cases (Dogu et al. 2007). ET cases had a median blood lead of 2.7 μg/dL, and controls had a median of 1.5 μg/dL. A 1-μg/dL increase in blood lead was associated with an OR for ET of 4.01 (95% CI: 2.53, 6.37). These results are in the same direction as the New York–based study, although the much stronger association could suggest some bias and highlights the need for further study in a prospective cohort setting. The age of study participants in the New York study was similar to those in our present study, but participants in the Turkish study were much younger (control mean, 51 ± 14 years). In our study population, the mean and median of blood lead concentrations were slightly higher than in the previous case–control studies, so exposure differences are not likely to explain the discrepant findings.

In another report, carriers of the *ALAD-2* allele showed a significantly larger odds of ET per unit increase in blood lead compared with those with *ALAD*-1-1 genotype (Louis et al. 2005). We did not find evidence for such an interaction for tremor scores. The *ALAD-2* protein binds more tightly to lead ions than the *ALAD-1* protein, although whether this confers greater toxicity to a given exposure to lead is unclear. In other studies in the NAS, interactions between lead and *ALAD* genotype have been seen in opposite directions for performance on a spatial copying task and mood symptoms (Rajan et al. 2007, 2008), whereas no interaction was seen for performance on several other cognitive tests (Rajan et al. 2008; Weuve et al. 2006).

Our study is the first to investigate the association between lead and tremor using bone lead concentration, a biomarker of cumulative lead exposure, and the first to explore this in a large cohort study. Nonetheless, there are limitations of our study that need to be considered as well. First, our findings were only among men, and thus may not be generalizable to women. Second, because high lead exposure is associated with various health ailments, it is possible that NAS participants with higher lead concentrations were less likely to participate in the bone lead or cognitive testing assessments. Thus, if the nonparticipation was also preferentially among men with more tremor, then our results would be biased downward. Perhaps most important, we used drawing samples to assess tremor severity, not a clinical examination. Although this tremor rating is not a clinical assessment of ET, it is an approach that has been used for prevalence estimation of ET (Hoffman et al. 2012), and we did find that tremor ratings increased with age as expected, validating to some extent our tremor assessment. However, not being a clinical assessment of ET, our tremor assessment may have been influenced by many factors other than ET itself. This would likely be more of a concern among older participants, and in fact we found results more in line with the prior ET case–control studies among the younger half of men in our sample (Louis 2008).

## Conclusions

Our results do not strongly support an association between lead exposure and tremor, and suggest no association with cumulative lead biomarkers. However, there is some suggestion for an association with blood lead when focusing on younger men. Further study in a cohort setting with lead biomarker data, but using clinical diagnoses of ET, are warranted.
